# Fifty years of the Canadian Field Epidemiology Program (1975–2025): Reflecting on the past, shaping the future

**DOI:** 10.14745/ccdr.v52i78a01

**Published:** 2026-07-30

**Authors:** Louis Wong, Anja Bilandzic, Kathleen Laberge

**Affiliations:** 1 Regulatory, Operations and Emergency Management Branch, Public Health Agency of Canada, Toronto, ON and Saint-Hyacinthe, QC

**Keywords:** public health surveillance, disease outbreak, epidemiology, health workforce

## Abstract

Since 1975, the Canadian Field Epidemiology Program (CFEP) has strengthened Canada’s public health capacity by training field epidemiologists through a two-year, service-oriented model that integrates formal learning with field placements and mentorship. Established at the request of the provinces and territories and modelled after the United States’ Epidemic Intelligence Service, CFEP has evolved into a nationally distributed surge and analytic capability embedded across jurisdictions.

Over five decades, trainees and alumni have supported outbreak and emergency response, strengthened surveillance systems, and produced applied evidence that has informed control measures and policy, spanning foodborne and environmental events, severe acute respiratory syndrome (SARS), H1N1, COVID-19, and emerging One Health threats.

The CFEP’s impact also reflects deliberate workforce development through structured mentorship, cohort-based learning, and alumni networks that extend expertise across federal, provincial, territorial, academic, and international settings.

As public health risks grow more complex due to climate-related hazards, rapid digital change, and challenges to trust, this commentary argues that CFEP must remain adaptive, deepen cross-sector partnerships, and embed equity, reconciliation, and systems thinking to sustain Canada’s health security over the next 50 years.

## Introduction

In 1975, the Government of Canada established the Canadian Field Epidemiology Program (CFEP) to strengthen public health capacity across the country. Created to build expertise in outbreak investigation and surveillance, CFEP has since become a key part of national health security infrastructure.

This thematic issue showcases the work of current CFEP trainees, illustrating how applied epidemiology continues to shape Canada’s response to complex health challenges. From infectious disease outbreaks to climate-related hazards, CFEP trainees have provided the expertise and agility that underpin our public health system. As we celebrate five decades of impact, we also ask: what does the next 50 years demand of us?

### Historical foundations and evolution

The CFEP, first known as the Canadian Field Epidemiology Training Program, was modelled after the United States’ Epidemic Intelligence Service and launched at the Laboratory Centre for Disease Control in 1975 (1) at the request of provinces and territories. It is designed as a two-year “learn-by-doing” training program, combining formal training with field placements embedded within provincial, territorial, and local health authorities (1,2). This “learn-by-doing” approach yields immediate system benefits, as trainees support public health priorities during training rather than only after program completion (3).

**Figure 1:**
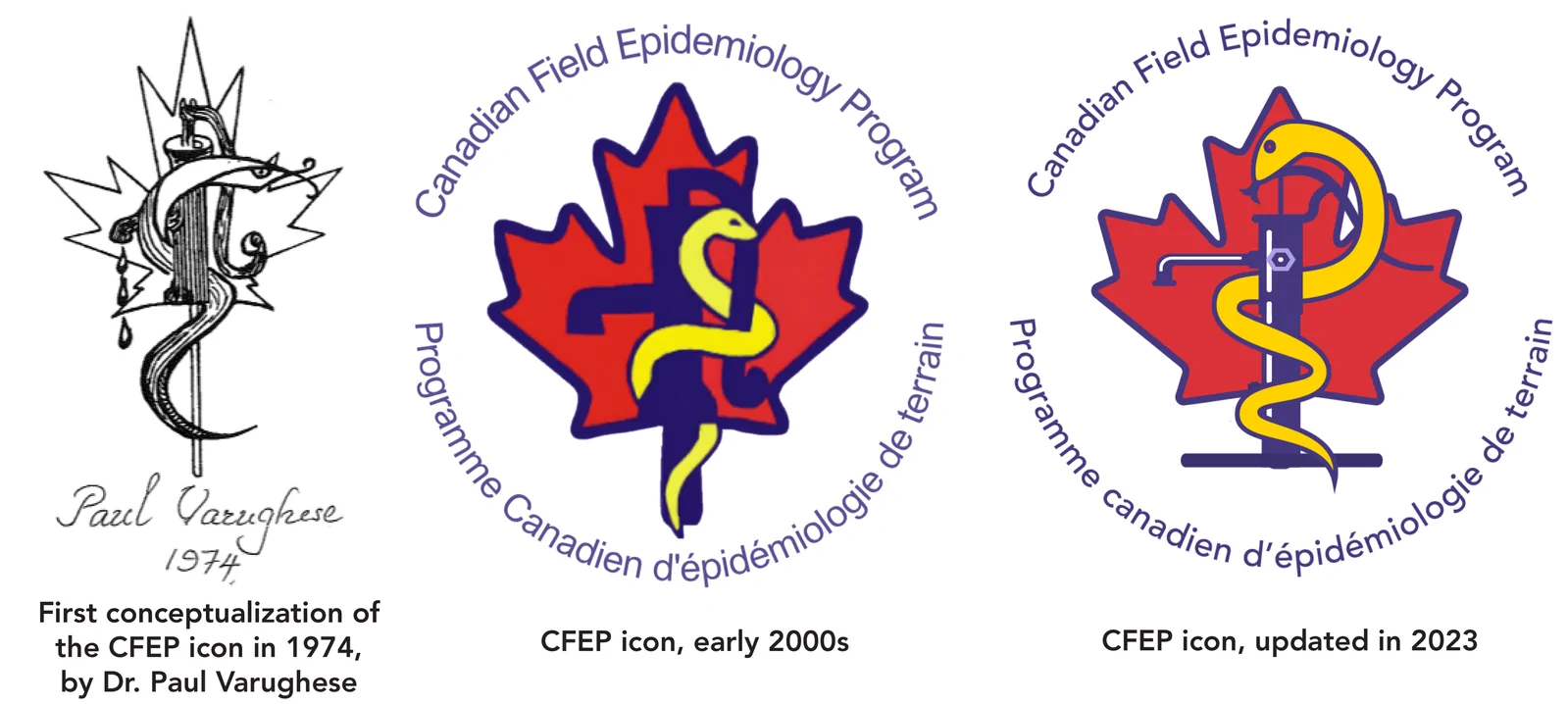
The evolution of the Canadian Field Epidemiology Program icon Abbreviation: CFEP, Canadian Field Epidemiology Program

From its inception, CFEP’s identity was symbolized by three key elements (**Figure 1**):

· The maple leaf, representing the program’s national scope

· The serpent from the staff of Asclepius, a universal symbol of health and medicine

· A water pump, a nod to John Snow’s historic removal of the Broad Street pump handle in 1854 to halt a cholera outbreak

In CFEP’s icon, the pump handle is restored, which signifies that modern field epidemiology is not only about removing hazards but also about restoring population health. This visual identity reflects the program’s longstanding mandate:

“To strengthen public health practice in Canada through the provision of training and service which foster excellence in the practice of applied epidemiology.”

In 1997, CFEP became a founding member of the Training Programs in Epidemiology and Public Health Interventions Network (TEPHINET), joining a global network of 84 field epidemiology training programs (4–6). Following the severe acute respiratory syndrome (SARS) outbreak in 2003, CFEP transitioned into the newly created Public Health Agency of Canada (PHAC) in 2004 (4), aligning its surge capacity with national emergency coordination. In 2016, CFEP earned TEPHINET accreditation, affirming its competency-based training standards against international benchmarks (4,5). In 2022, CFEP was reaccredited with Distinction and Merit in Emergency Response (5).

### Strategic contributions to public health

Over five decades, CFEP has delivered value across multiple dimensions of Canada’s public health system, as described below.

### Outbreak and emergency response

The CFEP trainees have been Canada’s backbone for rapid response during public health crises, establishing a practice-oriented ethos anchored in mentorship and rapid deployment. From foodborne outbreaks in the 1970s to SARS in 2003, H1N1 in 2009, and COVID-19 in 2020, CFEP trainees and alumni have provided surge capacity for case finding, contact tracing, epidemiologic analysis, and the establishment of surveillance systems (**Table 1**). Their work has informed control measures, guided policy decisions, and shaped national public health emergency protocols. Across successive public health challenges, CFEP trainees have remained central to Canada’s early detection and response efforts for emerging pathogens and issues.

**Table 1:** Examples of Canadian Field Epidemiology Program (CFEP) scientific communication products through the years

Year	Responses where CFEP was involved
1982	An epidemiologic assessment of unexplained deaths in a children’s hospital (7)
1987	Outbreak of toxic encephalopathy caused by eating mussels contaminated with domoic acid (8)
1997	A long, multinational *Salmonella* outbreak traced to a single widely distributed contaminated alfalfa seed lot, highlighting how modern food supply chains can rapidly spread illness across borders and why coordinated surveillance and stronger food safety controls are essential (9)
1998	A large 1997 Pacific Northwest outbreak linked to raw oysters across multiple jurisdictions showed how warmer coastal waters can amplify *Vibrio* risk and why strong, coordinated surveillance and rapid control actions (advisories/bed closures) are critical to prevent widespread illness (10)
2007	Canada’s first nosocomial outbreak of community-associated methicillin-resistant *Staphylococcus aureus* among healthy newborns and postpartum mothers in a Toronto maternal-newborn unit, underscoring the public health importance of rapid detection and strong infection-prevention controls to stop health care spread of emerging community strain (11)
2007	Use of a Web forum and an online questionnaire in the detection and investigation of an outbreak (12)
2015	Coordinated multi-province surveillance in Canada’s 2008 listeriosis outbreak traced illness to contaminated ready-to-eat deli meat and ongoing plant contamination, underscoring the need for enhanced listeriosis surveillance, tighter control of *Listeria* monocytogenes in ready-to-eat food facilities, and focused risk reduction guidance for institutions serving high risk groups (13)
2021	Identification of an unusual cluster of human granulocytic anaplasmosis in the Estrie region, Québec (14)
2021	A COVID-19 outbreak in a remote First Nations community in British Columbia linked to social gatherings and household transmission, which was effectively contained through a rapid, community-led public health response (15)

Abbreviation: CFEP, Canadian Field Epidemiology Program

As with comparable field epidemiology programs internationally, CFEP’s early emphasis reflected the dominant public health threats of the time: infectious disease detection and outbreak response (6,16,17). Today, the operating context is broadening. Trainees are increasingly mobilized to address syndemics and the cascading health impacts associated with climate change. This shift underscores the importance of maintaining an adaptable workforce capable of addressing complex, multi-hazard public health threats, while embracing the One Health approach, as demonstrated by a trainee’s contribution to the response to Canada’s first human case of highly pathogenic avian influenza.

### Public health surveillance system strengthening

The CFEP trainees assess, enhance and implement surveillance systems using standardized frameworks to ensure that Canada’s public health intelligence remains robust and fit for purpose. Recently, trainees worked on substance-related harms, sexually transmitted and blood-borne infections, heat-related illnesses, COVID-19, and numerous other topic areas that reflect the breadth of public health surveillance work across Canada.

Systems are often evaluated for the first time, resulting in actionable recommendations, and contributing to a legacy of quality improvement. These initiatives have led to improvements in communicable disease reporting, integration of laboratory data, and adoption of digital tools (18).

### Workforce development and networks

As of June 2026, CFEP has trained 239 field epidemiologists over its 50-year history. The CFEP alumni have become a driving force behind Canada’s public health leadership, shaping outbreak response, national policy, surveillance innovation, and strategic emergency management. Over the program’s history, CFEP graduates have advanced into some of the most influential public health roles in the country. Canada has had both a Chief Public Health Officer and a Deputy Chief Public Health Officer who are CFEP graduates. Beyond federal leadership, CFEP alumni have served as provincial Chief Medical Officers of Health, Chief Provincial/Territorial Epidemiologists, directors of public health intelligence and surveillance, and senior leaders across federal and provincial health ministries, local public health units, academia, and international organizations. Their expertise has informed national policy development, strengthened surveillance frameworks, and contributed to global health security.

A big part of this success comes from the mentorship built into the program. Trainees receive steady guidance from both CFEP staff and their placement site supervisors, which helps them grow their technical skills, navigate real-world investigations, and build confidence in their judgment. This support has been an important part of shaping the leaders that CFEP has produced.

The CFEP has also fostered collaboration between health systems and trusted colleagues through informal networks. Through strong connections within cohorts, among the broader alumni community, and throughout placement sites across Canada, CFEP trainees and graduates have become part of something greater than their individual training journeys. These informal networks mean that typically, someone “knows someone” when new public health situations arise, and this facilitates knowledge sharing opportunities. This function has been particularly useful when dealing with public health emergencies requiring unified action.

### Canadian Field Epidemiology Program today: Adapting to a changing landscape

**Figure 2:**
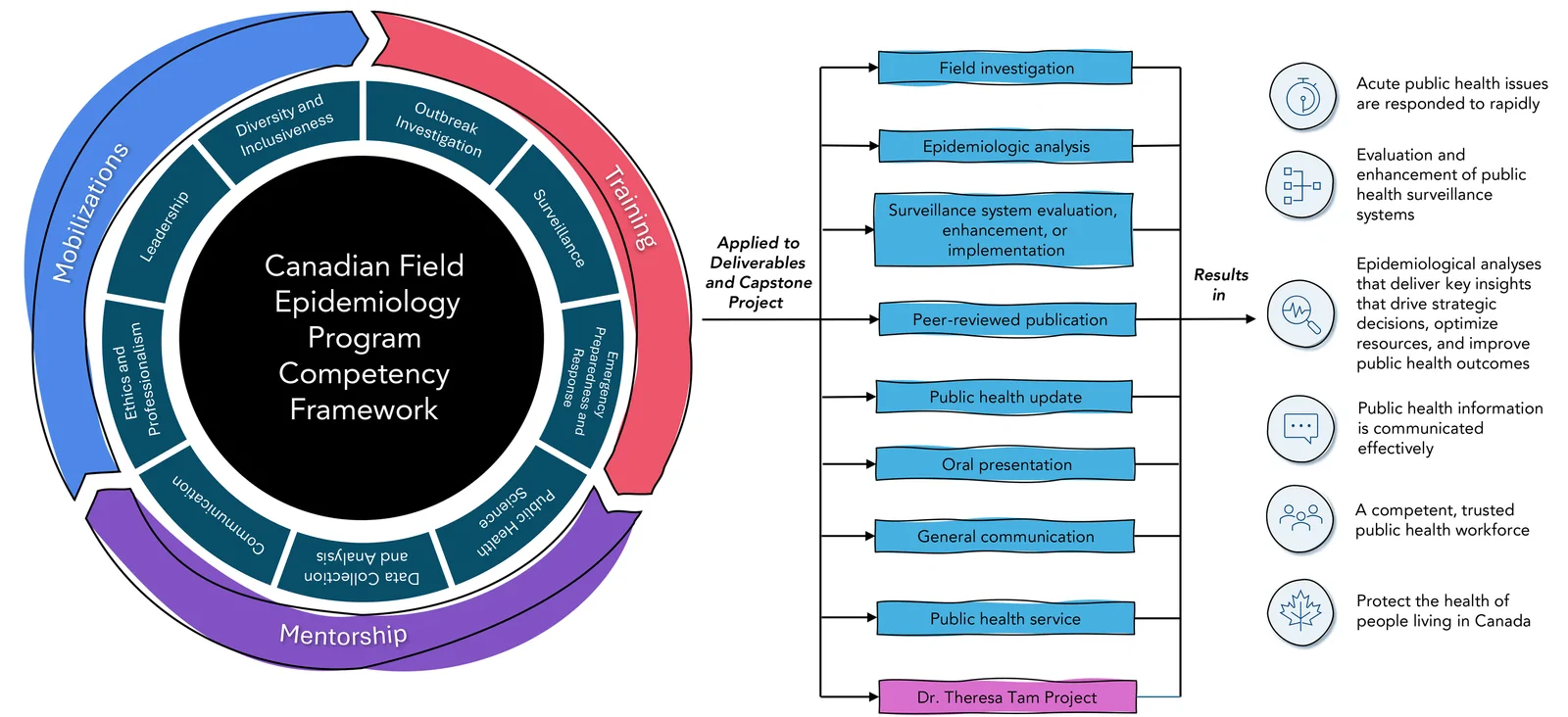
The Canadian Field Epidemiology Program model

The CFEP remains highly relevant in today’s complex health environment. Each year, it recruits a multidisciplinary cohort of five to ten trainees with backgrounds in epidemiology, public health and preventive medicine, nursing, veterinary sciences, and allied health, and places them in diverse settings for two years of immersive, service-oriented learning. The CFEP competency framework emphasizes outbreak investigation, epidemiologic analysis, surveillance evaluation, and communication, all of which are essential skills in public health practice (5). These competencies are reinforced by CFEP’s core values of excellence, integrity, collegiality, respect, adaptability, and impact, shaping not only what trainees learn, but how they work with partners and communities. The CFEP delivers its mandate through a competency-based training model (**Figure 2**). Trainees develop the necessary knowledge, skills, and professional attitudes through a standard curriculum, supervised deployments in response to public health events, and structured mentorship provided by both the program and placement sites.

### A vision for the future

A vision for the future begins with a clear understanding of what has changed. In today’s public health landscape, climate-related events are becoming more frequent and disruptive; emerging pathogens (e.g., COVID-19, mpox) and re-emerging pathogens (i.e., measles) are having devastating impacts, and surveillance and response tools are changing rapidly with advances in artificial intelligence. Equity and reconciliation are essential values that underpin effective public health action, and we have learned that trust in public health cannot be assumed.

A future-oriented CFEP should build on its strong foundation while continuing to adapt to the realities of public health. This means diversifying recruitment and training pathways to reflect the communities and contexts that public health serves, and strengthening interdisciplinary and cross-sector collaboration across public health, animal health, clinical care, laboratories, emergency management, social services, and community organizations. It also means reinforcing global partnerships and advancing the evolution of field epidemiology through a distinctly Canadian lens that reflects our geography, federal-provincial-territorial realities, and commitments to equity and Indigenous rights. Finally, future planning should emphasize leadership and systems thinking so that graduates can navigate complexity, respond to misinformation and mistrust, and support public health action that is timely, culturally safe, and effective.

### A foundational pillar for Canada’s public health

The CFEP is more than a training program; it is an engine of Canada’s public health capacity. For 50 years, it has helped Canada detect, investigate, and respond to health threats by developing skilled field epidemiologists and by contributing directly to surveillance and response efforts while trainees are still learning. The CFEP trainees and graduates have supported response operations, strengthened surveillance systems, and informed policies that protect communities across the country.

In the future, CFEP should deepen and formalize engagement with its graduates as a core asset for mentorship, recruitment, training placements, strengthening the training ecosystem, and extending the program’s reach. This is timely, as a CFEP alumni network is being established, creating a natural platform to support sustained connection, leadership development, and ongoing contributions to Canada’s public health capacity.

Looking ahead, the direction is clear: remain responsive to a shifting risk landscape and equip field epidemiologists to act early, lead confidently, and deliver effective public health action in complex systems. This issue honours CFEP’s legacy and highlights the work of current field epidemiologists, reaffirming the program’s commitment to remain agile, inclusive, and prepared for future health security challenges.
